# A quantitative linkage score for an association study following a linkage analysis

**DOI:** 10.1186/1471-2156-7-5

**Published:** 2006-01-20

**Authors:** Tao Wang, Robert C Elston

**Affiliations:** 1 Department of Epidemiology and Biostatistics, Case Western Reserve University, Cleveland, USA

## Abstract

**Background:**

Currently, a commonly used strategy for mapping complex quantitative traits is to use a genome-wide linkage analysis to narrow suspected genes to regions on a scale of centiMorgans (cM), followed by an association analysis to fine map the genetic variation in regions showing linkage. Two important questions arise in the design and the resulting inference at the association stage of this sequential procedure: (1) how should we design an efficient association study given the information provided by the previous linkage study? and (2) can an association in a linkage region explain, in part, the detected linkage signal?

**Results:**

We derive a quantitative linkage score (QLS) based on Haseman-Elston regression (Haseman and Elston 1972) and make use of this score to address both questions. In designing an association study, the selection of a subsample from the linkage study sample can be guided by the linkage information summarized in the QLS. When heterogeneity exists, we show that selection based on the QLS can increase the proportion of sample individuals from the subpopulation affected by a disease allele and therefore greatly improves the power of the association study. For the resulting inference, we frame as a hypothesis test the question of whether a linkage signal in a region can be in part explained by a marker allele. A simple one sided paired t-statistic is defined by comparing the two sets of QLSs obtained with/without modeling a marker association: a significant difference indicates that the marker can at least partly account for the detected linkage. We also show that this statistic can be used to detect a spurious association.

**Conclusion:**

All our results suggest that a careful examination of QLSs should be helpful for understanding the results of both association and linkage studies.

## Background

Identifying genes underlying complex quantitative traits, which are often heterogeneous and multifactorial, is still a great challenge in genetic epidemiology studies. Currently, a commonly used strategy for mapping complex traits is to use a genome-wide linkage analysis to narrow suspected genes to regions on a scale of centiMorgans (cM), followed by an association analysis to fine map the genetic variation in regions showing linkage. At the association stage of this sequential process, we are often interested in two questions: (1) how should we design a powerful and efficient association study given the information provided by the previous linkage study? and (2) can an association in a linkage region explain, in part, the detected linkage signal? Although these questions that arise respectively at the design and inference stages are two quite different aspects of an association study, they are related because both questions essentially rely on the interdependence of linkage and association. Here, we derive a quantitative linkage score (QLS) from Haseman-Elston linkage regression [1] and make use of this score to address both questions in the scenario of analyzing a complex quantitative trait.

The loci predisposing to a complex quantitative trait are usually expected to have small effects. One important reason for this, among others, is heterogeneity of the phenotype, where an allele of interest may have no effect on some individuals because they have different genetic and environmental backgrounds. If these individuals are included in the sample used in the association study, the effect of the examined allele is "diluted" and this leads to great difficulty in detecting association. Careful selection of individuals from the sample to exclude such possible "dilution" should presumably provide greater power. Ideally, we should like to find a variable, such as age, sex or ethnicity, that indicates heterogeneous persons. Unfortunately, such an indicator variable is often unclear or unavailable for a complex trait. Nevertheless, if an association study follows a linkage study, selection of the sample for the association study may be guided by the linkage information already obtained, using the linkage signal as a natural heterogeneity indicator. This idea has long been recognized and implemented in practice [2-4]. Fingerlin et al. (2004) systematically examined the selection of cases for a case-control association study based on allele-sharing information provided by affected members of a family [5]. We focus here on sample selection for an association study of a quantitative trait and show the usefulness of the QLS when heterogeneity exists.

After an association has been detected between the trait and a marker allele in the region of linkage, the question of whether this association accounts, in part, for the previously found linkage signal is not trivial. If the allele statistically associated with the trait is partly responsible for the linkage, we may be more confident that this allele is itself functional or in linkage disequilibrium with the true functional variant, rather than a false discovery resulting from other causes. On the other hand, if the associated allele cannot explain any linkage signal, we may consider adding more association markers to the region in order to avoid missing a possible genetic variant affecting the trait of interest. In the case of affected sibs (or other affected relatives) used for linkage analysis, one approach is to examine the difference in the allele sharing identical by descent (IBD) between members of families selected on the basis of the associated marker [2,6]. We address this question for a quantitative trait by testing whether there is a significant difference between the QLS with and without including this marker in the model. We show that this test is essentially the same as examining the interaction between the linkage and association signals and therefore is related to the genotype-IBD sharing test (GIST) proposed by Li et al. (2004) for affected sibship data[6]. Fulker (1999) proposed a similar idea, in the context of a variance component model, simultaneously modeling the association and linkage in the mean and variance-covariance structure of a family [7]. They focused on testing a similar, but different, hypothesis to determine whether the allele is the true candidate or is merely in disequilibrium with the trait locus, by comparing a model with all the parameters freely estimated to a model in which the linked genetic variance of the quantitative trait locus (QTL) is set to zero, on the assumption that there is a single variant responsible for the linkage signal[8].

In this paper, we propose a linkage score derived from quantitative trait linkage analysis that has important applications when an association study follows a linkage analysis. Although the linkage score derived here can be easily extended to general families, to implement our approach we focus here on nuclear families. We first derive the linkage score in the method section. Then we perform computer simulations to examine the usefulness of this score to select a sample for an association study when heterogeneity exists, and to clarify whether the association can, at least in part, explain the linkage signal.

## Methods

Our goal is to derive a score that captures the linkage information for quantitative traits in a way that will be useful for a follow-up association study. For simplicity of presentation, we assume the quantitative trait value may be affected by the presence of an allele without any other covariates present, which is not a necessary limitation for our derivation. We suppose linkage markers have been genotyped for family members and therefore the proportion of alleles shared IBD at a particular location can be estimated for all pairs of relatives in a pedigree [9,10].

### Quantitative linkage score (QLS)

We first derive the QLS. Suppose we have recruited *N *sibships. The trait value *y*_*ik *_of sib *i*(1, ..., *n*_*k*_) in sibship *k*(1, ..., *N*) is modeled by

*y*_*ik *_= *μ*_*k *_+ *x*_*ik*_*b *+*e*_*ik*_,     (1)

where *μ*_*k *_is the sibship specific mean, which absorbs family-level effects such as polygenic and common environmental effects [11]; *b *is the effect of the quantitative trait locus (QTL), which may include both additive and dominant effects; *x*_*ik *_is the corresponding vector of design variables indicating the genotype of the QTL; and *e*_*ik *_is an individual-level random effect. For simplicity of exposition only, we assume the QTL effect is additive and therefore *x*_*ik *_can be coded as one variable to indicate the number of copies of the allele of interest. Otherwise, it can be coded as a vector with two elements, for additive and dominant effects, respectively. Because in a linkage analysis the genotype of a QTL (*x*_*ik*_) is not observed (or the marker cannot be assumed to be in linkage disequilibrium with the QTL), we are not able to estimate directly. However, we can model the QTL effect in the variance-covariance matrix at the family-level. Under the trait model (1), the variance-covariance matrix of sibship *k *is given by

E((y1k−μk)(y1k−μk)…(y1k−μk)(ynkk−μk)………(y1k−μk)(ynkk−μk)…(ynkk−μk)(ynkk−μk))=(σb2+σe2…IBD1nkkσb2………IBD1nkkσb2…σb2+σe2),
 MathType@MTEF@5@5@+=feaafiart1ev1aaatCvAUfKttLearuWrP9MDH5MBPbIqV92AaeXatLxBI9gBaebbnrfifHhDYfgasaacH8akY=wiFfYdH8Gipec8Eeeu0xXdbba9frFj0=OqFfea0dXdd9vqai=hGuQ8kuc9pgc9s8qqaq=dirpe0xb9q8qiLsFr0=vr0=vr0dc8meaabaqaciaacaGaaeqabaqabeGadaaakeaacqWGfbqrdaqadaqaauaabeqadmaaaeaacqGGOaakcqWG5bqEdaWgaaWcbaGaeGymaeJaem4AaSgabeaakiabgkHiTGGaciab=X7aTnaaBaaaleaacqWGRbWAaeqaaOGaeiykaKIaeiikaGIaemyEaK3aaSbaaSqaaiabigdaXiabdUgaRbqabaGccqGHsislcqWF8oqBdaWgaaWcbaGaem4AaSgabeaakiabcMcaPaqaaiablAcilbqaaiabcIcaOiabdMha5naaBaaaleaacqaIXaqmcqWGRbWAaeqaaOGaeyOeI0Iae8hVd02aaSbaaSqaaiabdUgaRbqabaGccqGGPaqkcqGGOaakcqWG5bqEdaWgaaWcbaGaemOBa42aaSbaaWqaaiabdUgaRbqabaWccqWGRbWAaeqaaOGaeyOeI0Iae8hVd02aaSbaaSqaaiabdUgaRbqabaGccqGGPaqkaeaacqWIMaYsaeaacqWIMaYsaeaacqWIMaYsaeaacqGGOaakcqWG5bqEdaWgaaWcbaGaeGymaeJaem4AaSgabeaakiabgkHiTiab=X7aTnaaBaaaleaacqWGRbWAaeqaaOGaeiykaKIaeiikaGIaemyEaK3aaSbaaSqaaiabd6gaUnaaBaaameaacqWGRbWAaeqaaSGaem4AaSgabeaakiabgkHiTiab=X7aTnaaBaaaleaacqWGRbWAaeqaaOGaeiykaKcabaGaeSOjGSeabaGaeiikaGIaemyEaK3aaSbaaSqaaiabd6gaUnaaBaaameaacqWGRbWAaeqaaSGaem4AaSgabeaakiabgkHiTiab=X7aTnaaBaaaleaacqWGRbWAaeqaaOGaeiykaKIaeiikaGIaemyEaK3aaSbaaSqaaiabd6gaUnaaBaaameaacqWGRbWAaeqaaSGaem4AaSgabeaakiabgkHiTiab=X7aTnaaBaaaleaacqWGRbWAaeqaaOGaeiykaKcaaaGaayjkaiaawMcaaiabg2da9maabmaabaqbaeqabmWaaaqaaiab=n8aZnaaDaaaleaacqWGIbGyaeaacqaIYaGmaaGccqGHRaWkcqWFdpWCdaqhaaWcbaGaemyzaugabaGaeGOmaidaaaGcbaGaeSOjGSeabaGaemysaKKaemOqaiKaemiraq0aaSbaaSqaaiabigdaXiabd6gaUnaaBaaameaacqWGRbWAaeqaaSGaem4AaSgabeaakiab=n8aZnaaDaaaleaacqWGIbGyaeaacqaIYaGmaaaakeaacqWIMaYsaeaacqWIMaYsaeaacqWIMaYsaeaacqWGjbqscqWGcbGqcqWGebardaWgaaWcbaGaeGymaeJaemOBa42aaSbaaWqaaiabdUgaRbqabaWccqWGRbWAaeqaaOGae83Wdm3aa0baaSqaaiabdkgaIbqaaiabikdaYaaaaOqaaiablAcilbqaaiab=n8aZnaaDaaaleaacqWGIbGyaeaacqaIYaGmaaGccqGHRaWkcqWFdpWCdaqhaaWcbaGaemyzaugabaGaeGOmaidaaaaaaOGaayjkaiaawMcaaiabcYcaSaaa@C219@

where σb2
 MathType@MTEF@5@5@+=feaafiart1ev1aaatCvAUfKttLearuWrP9MDH5MBPbIqV92AaeXatLxBI9gBaebbnrfifHhDYfgasaacH8akY=wiFfYdH8Gipec8Eeeu0xXdbba9frFj0=OqFfea0dXdd9vqai=hGuQ8kuc9pgc9s8qqaq=dirpe0xb9q8qiLsFr0=vr0=vr0dc8meaabaqaciaacaGaaeqabaqabeGadaaakeaaiiGacqWFdpWCdaqhaaWcbaGaemOyaigabaGaeGOmaidaaaaa@30E2@ is the variance of the QTL, σe2
 MathType@MTEF@5@5@+=feaafiart1ev1aaatCvAUfKttLearuWrP9MDH5MBPbIqV92AaeXatLxBI9gBaebbnrfifHhDYfgasaacH8akY=wiFfYdH8Gipec8Eeeu0xXdbba9frFj0=OqFfea0dXdd9vqai=hGuQ8kuc9pgc9s8qqaq=dirpe0xb9q8qiLsFr0=vr0=vr0dc8meaabaqaciaacaGaaeqabaqabeGadaaakeaaiiGacqWFdpWCdaqhaaWcbaGaemyzaugabaGaeGOmaidaaaaa@30E8@ is the individual random effect variance and *IBD*_*ijk *_is the proportion of marker alleles shared IBD by sibs *i *and *j *in family *k*. Because both matrices are symmetric and the diagonal elements do not include linkage information, we only consider the lower triangular elements. We rearrange these elements of the above matrices as vectors of length *n*_*k*_(*n*_*k *_- 1)/2 by stacking one column on top of the other and then have

E((y1k−μk)(y2k−μk)…(yik−μk)(yjk−μk)..(y(n−1)kk−μk)(ynkk−μk)=(σb2IBD12k…σb2IBDijk…σb2IBD(n−1)nk).     (2)
 MathType@MTEF@5@5@+=feaafiart1ev1aaatCvAUfKttLearuWrP9MDH5MBPbIqV92AaeXatLxBI9gBaebbnrfifHhDYfgasaacH8akY=wiFfYdH8Gipec8Eeeu0xXdbba9frFj0=OqFfea0dXdd9vqai=hGuQ8kuc9pgc9s8qqaq=dirpe0xb9q8qiLsFr0=vr0=vr0dc8meaabaqaciaacaGaaeqabaqabeGadaaakeaacqWGfbqrdaqadaqaauaabeqafeaaaaqaaiabcIcaOiabdMha5naaBaaaleaacqaIXaqmcqWGRbWAaeqaaOGaeyOeI0ccciGae8hVd02aaSbaaSqaaiabdUgaRbqabaGccqGGPaqkcqGGOaakcqWG5bqEdaWgaaWcbaGaeGOmaiJaem4AaSgabeaakiabgkHiTiab=X7aTnaaBaaaleaacqWGRbWAaeqaaOGaeiykaKcabaGaeSOjGSeabaGaeiikaGIaemyEaK3aaSbaaSqaaiabdMgaPjabdUgaRbqabaGccqGHsislcqWF8oqBdaWgaaWcbaGaem4AaSgabeaakiabcMcaPiabcIcaOiabdMha5naaBaaaleaacqWGQbGAcqWGRbWAaeqaaOGaeyOeI0Iae8hVd02aaSbaaSqaaiabdUgaRbqabaGccqGGPaqkaeaacqGGUaGlcqGGUaGlaeaacqGGOaakcqWG5bqEdaWgaaWcbaGaeiikaGIaemOBa4MaeyOeI0IaeGymaeJaeiykaKYaaSbaaWqaaiabdUgaRbqabaWccqWGRbWAaeqaaOGaeyOeI0Iae8hVd02aaSbaaSqaaiabdUgaRbqabaGccqGGPaqkcqGGOaakcqWG5bqEdaWgaaWcbaGaemOBa42aaSbaaWqaaiabdUgaRbqabaWccqWGRbWAaeqaaOGaeyOeI0Iae8hVd02aaSbaaSqaaiabdUgaRbqabaaaaaGccaGLOaGaayzkaaGaeyypa0ZaaeWaaeaafaqabeqbbaaaaeaacqWFdpWCdaqhaaWcbaGaemOyaigabaGaeGOmaidaaOGaemysaKKaemOqaiKaemiraq0aaSbaaSqaaiabigdaXiabikdaYiabdUgaRbqabaaakeaacqWIMaYsaeaacqWFdpWCdaqhaaWcbaGaemOyaigabaGaeGOmaidaaOGaemysaKKaemOqaiKaemiraq0aaSbaaSqaaiabdMgaPjabdQgaQjabdUgaRbqabaaakeaacqWIMaYsaeaacqWFdpWCdaqhaaWcbaGaemOyaigabaGaeGOmaidaaOGaemysaKKaemOqaiKaemiraq0aaSbaaSqaaiabcIcaOiabd6gaUjabgkHiTiabigdaXiabcMcaPiabd6gaUjabdUgaRbqabaaaaaGccaGLOaGaayzkaaGaeiOla4IaaCzcaiaaxMaadaqadaqaaiabikdaYaGaayjkaiaawMcaaaaa@A4D8@

We can treat the above equation as a version of Haseman-Elston (HE) regression. The sibship specific mean *μ*_*k *_is usually unknown and needs to be estimated; various estimates have been discussed and a shrinkage estimate μ^k
 MathType@MTEF@5@5@+=feaafiart1ev1aaatCvAUfKttLearuWrP9MDH5MBPbIqV92AaeXatLxBI9gBaebbnrfifHhDYfgasaacH8akY=wiFfYdH8Gipec8Eeeu0xXdbba9frFj0=OqFfea0dXdd9vqai=hGuQ8kuc9pgc9s8qqaq=dirpe0xb9q8qiLsFr0=vr0=vr0dc8meaabaqaciaacaGaaeqabaqabeGadaaakeaaiiGacuWF8oqBgaqcamaaBaaaleaacqWGRbWAaeqaaaaa@3004@ has been recommended [11,12]. For the simulations performed in this paper, the μ^k
 MathType@MTEF@5@5@+=feaafiart1ev1aaatCvAUfKttLearuWrP9MDH5MBPbIqV92AaeXatLxBI9gBaebbnrfifHhDYfgasaacH8akY=wiFfYdH8Gipec8Eeeu0xXdbba9frFj0=OqFfea0dXdd9vqai=hGuQ8kuc9pgc9s8qqaq=dirpe0xb9q8qiLsFr0=vr0=vr0dc8meaabaqaciaacaGaaeqabaqabeGadaaakeaaiiGacuWF8oqBgaqcamaaBaaaleaacqWGRbWAaeqaaaaa@3004@ was estimated by the function *lme *in the *R *package . In a HE regression, linkage is detected by testing whether the QLT variance σb2
 MathType@MTEF@5@5@+=feaafiart1ev1aaatCvAUfKttLearuWrP9MDH5MBPbIqV92AaeXatLxBI9gBaebbnrfifHhDYfgasaacH8akY=wiFfYdH8Gipec8Eeeu0xXdbba9frFj0=OqFfea0dXdd9vqai=hGuQ8kuc9pgc9s8qqaq=dirpe0xb9q8qiLsFr0=vr0=vr0dc8meaabaqaciaacaGaaeqabaqabeGadaaakeaaiiGacqWFdpWCdaqhaaWcbaGaemOyaigabaGaeGOmaidaaaaa@30E2@ > 0, which is equivalent to testing the correlation between *IBD*_*ijk *_and the trait similarity between the two sibs, as measured by (*y*_*ik *_- μ^k
 MathType@MTEF@5@5@+=feaafiart1ev1aaatCvAUfKttLearuWrP9MDH5MBPbIqV92AaeXatLxBI9gBaebbnrfifHhDYfgasaacH8akY=wiFfYdH8Gipec8Eeeu0xXdbba9frFj0=OqFfea0dXdd9vqai=hGuQ8kuc9pgc9s8qqaq=dirpe0xb9q8qiLsFr0=vr0=vr0dc8meaabaqaciaacaGaaeqabaqabeGadaaakeaaiiGacuWF8oqBgaqcamaaBaaaleaacqWGRbWAaeqaaaaa@3004@)(*y*_*jk *_- μ^k
 MathType@MTEF@5@5@+=feaafiart1ev1aaatCvAUfKttLearuWrP9MDH5MBPbIqV92AaeXatLxBI9gBaebbnrfifHhDYfgasaacH8akY=wiFfYdH8Gipec8Eeeu0xXdbba9frFj0=OqFfea0dXdd9vqai=hGuQ8kuc9pgc9s8qqaq=dirpe0xb9q8qiLsFr0=vr0=vr0dc8meaabaqaciaacaGaaeqabaqabeGadaaakeaaiiGacuWF8oqBgaqcamaaBaaaleaacqWGRbWAaeqaaaaa@3004@) in our case. From this perspective, the linkage information provided by a sibpair can be captured by the score

Uijk=(yik−μ^k)(yjk−μ^k)(IBDijk−0.5)     (3)
 MathType@MTEF@5@5@+=feaafiart1ev1aaatCvAUfKttLearuWrP9MDH5MBPbIqV92AaeXatLxBI9gBaebbnrfifHhDYfgasaacH8akY=wiFfYdH8Gipec8Eeeu0xXdbba9frFj0=OqFfea0dXdd9vqai=hGuQ8kuc9pgc9s8qqaq=dirpe0xb9q8qiLsFr0=vr0=vr0dc8meaabaqaciaacaGaaeqabaqabeGadaaakeaacqWGvbqvdaWgaaWcbaGaemyAaKMaemOAaOMaem4AaSgabeaakiabg2da9iabcIcaOiabdMha5naaBaaaleaacqWGPbqAcqWGRbWAaeqaaOGaeyOeI0ccciGaf8hVd0MbaKaadaWgaaWcbaGaem4AaSgabeaakiabcMcaPiabcIcaOiabdMha5naaBaaaleaacqWGQbGAcqWGRbWAaeqaaOGaeyOeI0Iaf8hVd0MbaKaadaWgaaWcbaGaem4AaSgabeaakiabcMcaPiabcIcaOiabdMeajjabdkeacjabdseaenaaBaaaleaacqWGPbqAcqWGQbGAcqWGRbWAaeqaaOGaeyOeI0IaeGimaaJaeiOla4IaeGynauJaeiykaKIaaCzcaiaaxMaadaqadaqaaiabiodaZaGaayjkaiaawMcaaaaa@58B0@

From equation (3), we can see that for an additive trait model a positive score supports linkage and a negative score is evidence against linkage. When the inheritance model is unclear, we may take the "minmax" method to estimate the proportion of marker alleles shared IBD for a full sibpair, i.e. *IBD*_*ijk *_= 0.275*f*_*ijk*1_+ *f*_*ijk*2 _instead of *IBD*_*ijk *_= 0.5*f*_*ijk*1 _+ *f*_*ijk*2_, where *f*_*ijk*1 _and *f*_*ijk*2 _are probabilities of 1 and 2 alleles shared IBD, respectively [13]. We can simply sum the scores for all the pairs in a sibship to obtain a measure of linkage evidence for this sibship, because the sibship mean absorbs any residual correlation among the sibs. We may define the QLS more generally as *U*_*ijk*_*= S*_*ijk *_(*IBD*_*ijk *_- 0.5), where *S*_*ijk *_can be any measure of trait similarity, for example the squared sibpair difference, or a weighted average of the squared (mean-corrected) sum and the squared difference in trait values of two sibs, all of which are provided by different versions of HE regression implemented in the software SIBPAL of S.A.G.E. (2004). Different measures of trait similarity have been discussed in detail in the literature [e.g. [11,14-16]]. In those cases we may need to consider, in order to sum the QLSs within a sibship, a weight function appropriate for the correlation between scores among sibpairs. Note there is no difficulty in extending the QLS to qualitative traits. For example, for affected sibpairs *S*_*ijk *_can be defined as 1 for all pairs and the linkage score is simply given by *U*_*ijk *_= (*IBD*_*ijk *_- 0.5), which is related to the NPL score [17] and the statistic of the mean test [18].

### Application of the QLS in selecting a sample for an association study

We consider selecting a set of unrelated individuals from sibships previously used for a QTL linkage analysis. In the case of a complex quantitative trait where heterogeneity exists, the goal of an association study is to detect a variant with maximum power. We emphasize that such a study would not be a classic epidemiologlcal study done to determine the attributable risk, for which subjects should be drawn randomly from a population. Rather, the study we discuss here is done for gene finding and therefore the selection of the sample should be done to provide maximum power rather than to represent the whole population.

Suppose that a population consists of two subpopulations (P1 and P2) with proportions *q*_1 _and *q*_2 _respectively (*q*_1 _+ *q*_2 _= 1), where the gene variant has an effect in only one subpopulation (P1). To examine the usefulness of the QLS in selecting a sample for an association study, we theoretically compare the proportions of individuals affected by a disease allele selected from a homogenous subpopulation (P1) in two selected samples: one sample is obtained by randomly selecting sibships (proportion *q*_*r*_) and the other is obtained by selecting sibships with QLS>0 (proportion *q*_*qls*_). To simplify the theoretical derivation, we assume known IBD sharing and sibships of size 2 (independent sibpairs).

Let *T*_*k *_= [(*y*_1*k *_- *μ_k_*), (*y*_2*k *_- *μ_k_*)]^*T*^, where the superscript *T *denotes transpose and the subscripts 1 and 2 indicate two sibs in a sibship. With the assumption of normal individual effects e_*ik*_, *T *~ *N*(0, Σ_*k*_), where

∑k=[(σb2+σe2)IBD12kσb2IBD12kσb2(σb2+σe2)].
 MathType@MTEF@5@5@+=feaafiart1ev1aaatCvAUfKttLearuWrP9MDH5MBPbIqV92AaeXatLxBI9gBaebbnrfifHhDYfgasaacH8akY=wiFfYdH8Gipec8Eeeu0xXdbba9frFj0=OqFfea0dXdd9vqai=hGuQ8kuc9pgc9s8qqaq=dirpe0xb9q8qiLsFr0=vr0=vr0dc8meaabaqaciaacaGaaeqabaqabeGadaaakeaadaaeqaqaaiabg2da9maadmaabaqbaeqabiGaaaqaaiabcIcaOGGaciab=n8aZnaaDaaaleaacqWGIbGyaeaacqaIYaGmaaGccqGHRaWkcqWFdpWCdaqhaaWcbaGaemyzaugabaGaeGOmaidaaOGaeiykaKcabaGaemysaKKaemOqaiKaemiraq0aaSbaaSqaaiabigdaXiabikdaYiabdUgaRbqabaGccqWFdpWCdaqhaaWcbaGaemOyaigabaGaeGOmaidaaaGcbaGaemysaKKaemOqaiKaemiraq0aaSbaaSqaaiabigdaXiabikdaYiabdUgaRbqabaGccqWFdpWCdaqhaaWcbaGaemOyaigabaGaeGOmaidaaaGcbaGaeiikaGIae83Wdm3aa0baaSqaaiabdkgaIbqaaiabikdaYaaakiabgUcaRiab=n8aZnaaDaaaleaacqWGLbqzaeaacqaIYaGmaaGccqGGPaqkaaaacaGLBbGaayzxaaaaleaacqWGRbWAaeqaniabggHiLdGccqGGUaGlaaa@5FBC@

**Figure 1:**
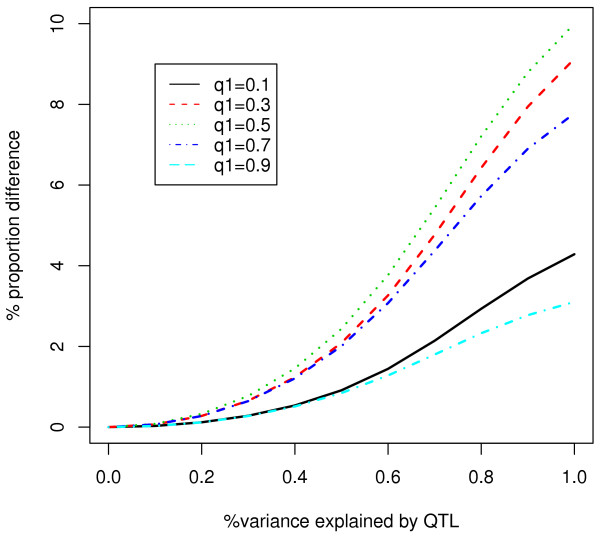
**Difference in the proportion of individuals from subpopulation 1 between random sampling and QLS sampling**. Subpopulation 1 comprises people who are affected by the QTL and *q*_1 _is the proportion of subpopulation 1 in the whole population.

To further simplify the presentation, we standardize *T*_*k *_as Z_*k*_, so that the correlation matrix of *Z*_*k *_is

(1ρkρk1)
 MathType@MTEF@5@5@+=feaafiart1ev1aaatCvAUfKttLearuWrP9MDH5MBPbIqV92AaeXatLxBI9gBaebbnrfifHhDYfgasaacH8akY=wiFfYdH8Gipec8Eeeu0xXdbba9frFj0=OqFfea0dXdd9vqai=hGuQ8kuc9pgc9s8qqaq=dirpe0xb9q8qiLsFr0=vr0=vr0dc8meaabaqaciaacaGaaeqabaqabeGadaaakeaadaqadaqaauaabeqaciaaaeaacqaIXaqmaeaaiiGacqWFbpGCdaWgaaWcbaGaem4AaSgabeaaaOqaaiab=f8aYnaaBaaaleaacqWGRbWAaeqaaaGcbaGaeGymaedaaaGaayjkaiaawMcaaaaa@36D1@

where *ρ*_*k *_= 0, 0.5σb2
 MathType@MTEF@5@5@+=feaafiart1ev1aaatCvAUfKttLearuWrP9MDH5MBPbIqV92AaeXatLxBI9gBaebbnrfifHhDYfgasaacH8akY=wiFfYdH8Gipec8Eeeu0xXdbba9frFj0=OqFfea0dXdd9vqai=hGuQ8kuc9pgc9s8qqaq=dirpe0xb9q8qiLsFr0=vr0=vr0dc8meaabaqaciaacaGaaeqabaqabeGadaaakeaaiiGacqWFdpWCdaqhaaWcbaGaemOyaigabaGaeGOmaidaaaaa@30E2@/(σb2
 MathType@MTEF@5@5@+=feaafiart1ev1aaatCvAUfKttLearuWrP9MDH5MBPbIqV92AaeXatLxBI9gBaebbnrfifHhDYfgasaacH8akY=wiFfYdH8Gipec8Eeeu0xXdbba9frFj0=OqFfea0dXdd9vqai=hGuQ8kuc9pgc9s8qqaq=dirpe0xb9q8qiLsFr0=vr0=vr0dc8meaabaqaciaacaGaaeqabaqabeGadaaakeaaiiGacqWFdpWCdaqhaaWcbaGaemOyaigabaGaeGOmaidaaaaa@30E2@ + σe2
 MathType@MTEF@5@5@+=feaafiart1ev1aaatCvAUfKttLearuWrP9MDH5MBPbIqV92AaeXatLxBI9gBaebbnrfifHhDYfgasaacH8akY=wiFfYdH8Gipec8Eeeu0xXdbba9frFj0=OqFfea0dXdd9vqai=hGuQ8kuc9pgc9s8qqaq=dirpe0xb9q8qiLsFr0=vr0=vr0dc8meaabaqaciaacaGaaeqabaqabeGadaaakeaaiiGacqWFdpWCdaqhaaWcbaGaemyzaugabaGaeGOmaidaaaaa@30E8@) and σb2
 MathType@MTEF@5@5@+=feaafiart1ev1aaatCvAUfKttLearuWrP9MDH5MBPbIqV92AaeXatLxBI9gBaebbnrfifHhDYfgasaacH8akY=wiFfYdH8Gipec8Eeeu0xXdbba9frFj0=OqFfea0dXdd9vqai=hGuQ8kuc9pgc9s8qqaq=dirpe0xb9q8qiLsFr0=vr0=vr0dc8meaabaqaciaacaGaaeqabaqabeGadaaakeaaiiGacqWFdpWCdaqhaaWcbaGaemOyaigabaGaeGOmaidaaaaa@30E2@/(σb2
 MathType@MTEF@5@5@+=feaafiart1ev1aaatCvAUfKttLearuWrP9MDH5MBPbIqV92AaeXatLxBI9gBaebbnrfifHhDYfgasaacH8akY=wiFfYdH8Gipec8Eeeu0xXdbba9frFj0=OqFfea0dXdd9vqai=hGuQ8kuc9pgc9s8qqaq=dirpe0xb9q8qiLsFr0=vr0=vr0dc8meaabaqaciaacaGaaeqabaqabeGadaaakeaaiiGacqWFdpWCdaqhaaWcbaGaemOyaigabaGaeGOmaidaaaaa@30E2@ + σe2
 MathType@MTEF@5@5@+=feaafiart1ev1aaatCvAUfKttLearuWrP9MDH5MBPbIqV92AaeXatLxBI9gBaebbnrfifHhDYfgasaacH8akY=wiFfYdH8Gipec8Eeeu0xXdbba9frFj0=OqFfea0dXdd9vqai=hGuQ8kuc9pgc9s8qqaq=dirpe0xb9q8qiLsFr0=vr0=vr0dc8meaabaqaciaacaGaaeqabaqabeGadaaakeaaiiGacqWFdpWCdaqhaaWcbaGaemyzaugabaGaeGOmaidaaaaa@30E8@), respectively, for proportions 0, 0.5, and 1 allele sharing IBD. With the assumption that a random sample of sibpairs is used for the linkage analysis, we have *q*_*r *_= *q*_1 _and

qqls=q1q1+q2[1π arctan (ρIBD=121−ρIBD=12)+1],     (4)
 MathType@MTEF@5@5@+=feaafiart1ev1aaatCvAUfKttLearuWrP9MDH5MBPbIqV92AaeXatLxBI9gBaebbnrfifHhDYfgasaacH8akY=wiFfYdH8Gipec8Eeeu0xXdbba9frFj0=OqFfea0dXdd9vqai=hGuQ8kuc9pgc9s8qqaq=dirpe0xb9q8qiLsFr0=vr0=vr0dc8meaabaqaciaacaGaaeqabaqabeGadaaakeaacqWGXbqCdaWgaaWcbaGaemyCaeNaemiBaWMaem4Camhabeaakiabg2da9maalaaabaGaemyCae3aaSbaaSqaaiabigdaXaqabaaakeaacqWGXbqCdaWgaaWcbaGaeGymaedabeaakiabgUcaRmaalaaabaGaemyCae3aaSbaaSqaaiabikdaYaqabaaakeaadaWadaqaamaaleaaleaacqaIXaqmaeaaiiGacqWFapaCaaGccqqGGaaicqqGHbqycqqGYbGCcqqGJbWycqqG0baDcqqGHbqycqqGUbGBcqqGGaaidaqadaqaamaaleaaleaacqWFbpGCdaqhaaadbaGaemysaKKaemOqaiKaemiraqKaeyypa0JaeGymaedabaGaeGOmaidaaaWcbaGaeGymaeJaeyOeI0Iae8xWdi3aa0baaWqaaiabdMeajjabdkeacjabdseaejabg2da9iabigdaXaqaaiabikdaYaaaaaaakiaawIcacaGLPaaacqGHRaWkcqaIXaqmaiaawUfacaGLDbaaaaaaaiabcYcaSiaaxMaacaWLjaWaaeWaaeaacqaI0aanaiaawIcacaGLPaaaaaa@64DB@

where *ρ*_*IBD *= 1 _is the correlation between two sibs of a pair with proportion 1 IBD sharing. (see Appendix 1). It is obvious that [1π arctan (ρIBD=121−ρIBD=12+1)]≥1
 MathType@MTEF@5@5@+=feaafiart1ev1aaatCvAUfKttLearuWrP9MDH5MBPbIqV92AaeXatLxBI9gBaebbnrfifHhDYfgasaacH8akY=wiFfYdH8Gipec8Eeeu0xXdbba9frFj0=OqFfea0dXdd9vqai=hGuQ8kuc9pgc9s8qqaq=dirpe0xb9q8qiLsFr0=vr0=vr0dc8meaabaqaciaacaGaaeqabaqabeGadaaakeaadaWadaqaamaalaaabaGaeGymaedabaacciGae8hWdahaaiabbccaGiabbggaHjabbkhaYjabbogaJjabbsha0jabbggaHjabb6gaUjabbccaGmaabmaabaWaaSaaaeaacqWFbpGCdaqhaaWcbaGaemysaKKaemOqaiKaemiraqKaeyypa0JaeGymaedabaGaeGOmaidaaaGcbaGaeGymaeJaeyOeI0Iae8xWdi3aa0baaSqaaiabdMeajjabdkeacjabdseaejabg2da9iabigdaXaqaaiabikdaYaaaaaGccqGHRaWkcqaIXaqmaiaawIcacaGLPaaaaiaawUfacaGLDbaacqGHLjYScqaIXaqmaaa@5330@ , and so *q*_*qls *_is always ≥ *q*_*r*_. From this inequality, we can also see that the difference between *q*_*qls *_and *q*_*r *_depends on (1) the proportion of P1: when *q*_1 _= 0.5, the difference is maximum; and (2) the variance explained by the QTL: the difference is an increasing function of *ρ*_*IBD *= 1_, i.e. σb2
 MathType@MTEF@5@5@+=feaafiart1ev1aaatCvAUfKttLearuWrP9MDH5MBPbIqV92AaeXatLxBI9gBaebbnrfifHhDYfgasaacH8akY=wiFfYdH8Gipec8Eeeu0xXdbba9frFj0=OqFfea0dXdd9vqai=hGuQ8kuc9pgc9s8qqaq=dirpe0xb9q8qiLsFr0=vr0=vr0dc8meaabaqaciaacaGaaeqabaqabeGadaaakeaaiiGacqWFdpWCdaqhaaWcbaGaemOyaigabaGaeGOmaidaaaaa@30E2@/(σb2
 MathType@MTEF@5@5@+=feaafiart1ev1aaatCvAUfKttLearuWrP9MDH5MBPbIqV92AaeXatLxBI9gBaebbnrfifHhDYfgasaacH8akY=wiFfYdH8Gipec8Eeeu0xXdbba9frFj0=OqFfea0dXdd9vqai=hGuQ8kuc9pgc9s8qqaq=dirpe0xb9q8qiLsFr0=vr0=vr0dc8meaabaqaciaacaGaaeqabaqabeGadaaakeaaiiGacqWFdpWCdaqhaaWcbaGaemOyaigabaGaeGOmaidaaaaa@30E2@ + σe2
 MathType@MTEF@5@5@+=feaafiart1ev1aaatCvAUfKttLearuWrP9MDH5MBPbIqV92AaeXatLxBI9gBaebbnrfifHhDYfgasaacH8akY=wiFfYdH8Gipec8Eeeu0xXdbba9frFj0=OqFfea0dXdd9vqai=hGuQ8kuc9pgc9s8qqaq=dirpe0xb9q8qiLsFr0=vr0=vr0dc8meaabaqaciaacaGaaeqabaqabeGadaaakeaaiiGacqWFdpWCdaqhaaWcbaGaemyzaugabaGaeGOmaidaaaaa@30E8@). The difference between *q*_*qls *_and *q*_*r *_is presented in Figure 1, which shows that selection based on the QLS can increase the proportion of individuals from subpopulation 1 at most 10%. Nevertheless, a slight difference in this proportion is not trivial, because it may greatly improve the power of an association study (see results).

### Application of the QLS to assess the correlation of association with previous linkage

To answer the question of whether a linkage signal in a region can be in part explained by a marker allele used in an association study, we compare the QLS on incorporating and not incorporating this marker into the trait model (equation 1), which we call the first (or individual) level regression, to distinguish it from the second (or family) level regression (equation 2). We frame this problem as a hypothesis test. When a marker is included in the model at the individual level, the variance-covariance matrix of sibship *k *is given by

E((y1k−μk−x1kb)(y1k−μk−x1kb)…(y1k−μk−x1kb)(ynkk−μk−xnkkb)⋯⋯⋯(y1k−μk−x1kb)(ynkk−μk−xnkkb)⋯(ynkk−μk−xnkkb)(ynkk−μk−xnkkb))=(σb2+σe2…IBD1nkkσb2………IBD1nkkσb2…σb2+σe2)
 MathType@MTEF@5@5@+=feaafiart1ev1aaatCvAUfKttLearuWrP9MDH5MBPbIqV92AaeXatLxBI9gBaebbnrfifHhDYfgasaacH8akY=wiFfYdH8Gipec8Eeeu0xXdbba9frFj0=OqFfea0dXdd9vqai=hGuQ8kuc9pgc9s8qqaq=dirpe0xb9q8qiLsFr0=vr0=vr0dc8meaabaqaciaacaGaaeqabaqabeGadaaakeaafaqabeGabaaabaGaemyrau0aaeWaaeaafaqabeWadaaabaGaeiikaGIaemyEaK3aaSbaaSqaaiabigdaXiabdUgaRbqabaGccqGHsisliiGacqWF8oqBdaWgaaWcbaGaem4AaSgabeaakiabgkHiTiabdIha4naaBaaaleaacqaIXaqmcqWGRbWAaeqaaOGaemOyaiMaeiykaKIaeiikaGIaemyEaK3aaSbaaSqaaiabigdaXiabdUgaRbqabaGccqGHsislcqWF8oqBdaWgaaWcbaGaem4AaSgabeaakiabgkHiTiabdIha4naaBaaaleaacqaIXaqmcqWGRbWAaeqaaOGaemOyaiMaeiykaKcabaGaeSOjGSeabaGaeiikaGIaemyEaK3aaSbaaSqaaiabigdaXiabdUgaRbqabaGccqGHsislcqWF8oqBdaWgaaWcbaGaem4AaSgabeaakiabgkHiTiabdIha4naaBaaaleaacqaIXaqmcqWGRbWAaeqaaOGaemOyaiMaeiykaKIaeiikaGIaemyEaK3aaSbaaSqaaiabd6gaUnaaBaaameaacqWGRbWAaeqaaSGaem4AaSgabeaakiabgkHiTiab=X7aTnaaBaaaleaacqWGRbWAaeqaaOGaeyOeI0IaemiEaG3aaSbaaSqaaiabd6gaUnaaBaaameaacqWGRbWAaeqaaSGaem4AaSgabeaakiabdkgaIjabcMcaPaqaaiabl+Uimbqaaiabl+Uimbqaaiabl+UimbqaaiabcIcaOiabdMha5naaBaaaleaacqaIXaqmcqWGRbWAaeqaaOGaeyOeI0Iae8hVd02aaSbaaSqaaiabdUgaRbqabaGccqGHsislcqWG4baEdaWgaaWcbaGaeGymaeJaem4AaSgabeaakiabdkgaIjabcMcaPiabcIcaOiabdMha5naaBaaaleaacqWGUbGBdaWgaaadbaGaem4AaSgabeaaliabdUgaRbqabaGccqGHsislcqWF8oqBdaWgaaWcbaGaem4AaSgabeaakiabgkHiTiabdIha4naaBaaaleaacqWGUbGBdaWgaaadbaGaem4AaSgabeaaliabdUgaRbqabaGccqWGIbGycqGGPaqkaeaacqWIVlctaeaacqGGOaakcqWG5bqEdaWgaaWcbaGaemOBa42aaSbaaWqaaiabdUgaRbqabaWccqWGRbWAaeqaaOGaeyOeI0Iae8hVd02aaSbaaSqaaiabdUgaRbqabaGccqGHsislcqWG4baEdaWgaaWcbaGaemOBa42aaSbaaWqaaiabdUgaRbqabaWccqWGRbWAaeqaaOGaemOyaiMaeiykaKIaeiikaGIaemyEaK3aaSbaaSqaaiabd6gaUnaaBaaameaacqWGRbWAaeqaaSGaem4AaSgabeaakiabgkHiTiab=X7aTnaaBaaaleaacqWGRbWAaeqaaOGaeyOeI0IaemiEaG3aaSbaaSqaaiabd6gaUnaaBaaameaacqWGRbWAaeqaaSGaem4AaSgabeaakiabdkgaIjabcMcaPaaaaiaawIcacaGLPaaaaeaacqGH9aqpdaqadaqaauaabeqadmaaaeaacqWFdpWCdaqhaaWcbaGaemOyaigabaGaeGOmaidaaOGaey4kaSIae83Wdm3aa0baaSqaaiabdwgaLbqaaiabikdaYaaaaOqaaiablAcilbqaaiabdMeajjabdkeacjabdseaenaaBaaaleaacqaIXaqmcqWGUbGBdaWgaaadbaGaem4AaSgabeaaliabdUgaRbqabaGccqWFdpWCdaqhaaWcbaGaemOyaigabaGaeGOmaidaaaGcbaGaeSOjGSeabaGaeSOjGSeabaGaeSOjGSeabaGaemysaKKaemOqaiKaemiraq0aaSbaaSqaaiabigdaXiabd6gaUnaaBaaameaacqWGRbWAaeqaaSGaem4AaSgabeaakiab=n8aZnaaDaaaleaacqWGIbGyaeaacqaIYaGmaaaakeaacqWIMaYsaeaacqWFdpWCdaqhaaWcbaGaemOyaigabaGaeGOmaidaaOGaey4kaSIae83Wdm3aa0baaSqaaiabdwgaLbqaaiabikdaYaaaaaaakiaawIcacaGLPaaaaaaaaa@FE66@

where *x*_*ik *_is a genotype code for the marker and *b *is its effect on the trait, which may arise from a "true" association (the marker is the QTL itself or is in linkage disequilibrium with the QTL), or from a "spurious" association (e.g. due to population stratification). Based on the above equation, we can obtain the corresponding QLS with the marker included in the above regression model, which is given by

Uijk=(yik−μ^k−xikb^)(yjk−μ^k−xjkb^)(IBDijk−0.5),
 MathType@MTEF@5@5@+=feaafiart1ev1aaatCvAUfKttLearuWrP9MDH5MBPbIqV92AaeXatLxBI9gBaebbnrfifHhDYfgasaacH8akY=wiFfYdH8Gipec8Eeeu0xXdbba9frFj0=OqFfea0dXdd9vqai=hGuQ8kuc9pgc9s8qqaq=dirpe0xb9q8qiLsFr0=vr0=vr0dc8meaabaqaciaacaGaaeqabaqabeGadaaakeaacqWGvbqvdaWgaaWcbaGaemyAaKMaemOAaOMaem4AaSgabeaakiabg2da9iabcIcaOiabdMha5naaBaaaleaacqWGPbqAcqWGRbWAaeqaaOGaeyOeI0ccciGaf8hVd0MbaKaadaWgaaWcbaGaem4AaSgabeaakiabgkHiTiabdIha4naaBaaaleaacqWGPbqAcqWGRbWAaeqaaOGafmOyaiMbaKaacqGGPaqkcqGGOaakcqWG5bqEdaWgaaWcbaGaemOAaOMaem4AaSgabeaakiabgkHiTiqb=X7aTzaajaWaaSbaaSqaaiabdUgaRbqabaGccqGHsislcqWG4baEdaWgaaWcbaGaemOAaOMaem4AaSgabeaakiqbdkgaIzaajaGaeiykaKIaeiikaGIaemysaKKaemOqaiKaemiraq0aaSbaaSqaaiabdMgaPjabdQgaQjabdUgaRbqabaGccqGHsislcqaIWaamcqGGUaGlcqaI1aqncqGGPaqkcqGGSaalaaa@6337@

where b^
 MathType@MTEF@5@5@+=feaafiart1ev1aaatCvAUfKttLearuWrP9MDH5MBPbIqV92AaeXatLxBI9gBaebbnrfifHhDYfgasaacH8akY=wiFfYdH8Gipec8Eeeu0xXdbba9frFj0=OqFfea0dXdd9vqai=hGuQ8kuc9pgc9s8qqaq=dirpe0xb9q8qiLsFr0=vr0=vr0dc8meaabaqaciaacaGaaeqabaqabeGadaaakeaacuWGIbGygaqcaaaa@2E09@ and μ^k
 MathType@MTEF@5@5@+=feaafiart1ev1aaatCvAUfKttLearuWrP9MDH5MBPbIqV92AaeXatLxBI9gBaebbnrfifHhDYfgasaacH8akY=wiFfYdH8Gipec8Eeeu0xXdbba9frFj0=OqFfea0dXdd9vqai=hGuQ8kuc9pgc9s8qqaq=dirpe0xb9q8qiLsFr0=vr0=vr0dc8meaabaqaciaacaGaaeqabaqabeGadaaakeaaiiGacuWF8oqBgaqcamaaBaaaleaacqWGRbWAaeqaaaaa@3004@ are the estimates of *b *and *μ_k_*, respectively. In the following presentation, we denote the QLS obtained with and without modeling an association marker Uijk(a)
 MathType@MTEF@5@5@+=feaafiart1ev1aaatCvAUfKttLearuWrP9MDH5MBPbIqV92AaeXatLxBI9gBaebbnrfifHhDYfgasaacH8akY=wiFfYdH8Gipec8Eeeu0xXdbba9frFj0=OqFfea0dXdd9vqai=hGuQ8kuc9pgc9s8qqaq=dirpe0xb9q8qiLsFr0=vr0=vr0dc8meaabaqaciaacaGaaeqabaqabeGadaaakeaacqWGvbqvdaqhaaWcbaGaemyAaKMaemOAaOMaem4AaSgabaGaeiikaGIaemyyaeMaeiykaKcaaaaa@3520@ and Uijk(b)
 MathType@MTEF@5@5@+=feaafiart1ev1aaatCvAUfKttLearuWrP9MDH5MBPbIqV92AaeXatLxBI9gBaebbnrfifHhDYfgasaacH8akY=wiFfYdH8Gipec8Eeeu0xXdbba9frFj0=OqFfea0dXdd9vqai=hGuQ8kuc9pgc9s8qqaq=dirpe0xb9q8qiLsFr0=vr0=vr0dc8meaabaqaciaacaGaaeqabaqabeGadaaakeaacqWGvbqvdaqhaaWcbaGaemyAaKMaemOAaOMaem4AaSgabaGaeiikaGIaemOyaiMaeiykaKcaaaaa@3522@, respectively. Given these two sets of QLSs, Uijk(a)
 MathType@MTEF@5@5@+=feaafiart1ev1aaatCvAUfKttLearuWrP9MDH5MBPbIqV92AaeXatLxBI9gBaebbnrfifHhDYfgasaacH8akY=wiFfYdH8Gipec8Eeeu0xXdbba9frFj0=OqFfea0dXdd9vqai=hGuQ8kuc9pgc9s8qqaq=dirpe0xb9q8qiLsFr0=vr0=vr0dc8meaabaqaciaacaGaaeqabaqabeGadaaakeaacqWGvbqvdaqhaaWcbaGaemyAaKMaemOAaOMaem4AaSgabaGaeiikaGIaemyyaeMaeiykaKcaaaaa@3520@ and Uijk(b)
 MathType@MTEF@5@5@+=feaafiart1ev1aaatCvAUfKttLearuWrP9MDH5MBPbIqV92AaeXatLxBI9gBaebbnrfifHhDYfgasaacH8akY=wiFfYdH8Gipec8Eeeu0xXdbba9frFj0=OqFfea0dXdd9vqai=hGuQ8kuc9pgc9s8qqaq=dirpe0xb9q8qiLsFr0=vr0=vr0dc8meaabaqaciaacaGaaeqabaqabeGadaaakeaacqWGvbqvdaqhaaWcbaGaemyAaKMaemOAaOMaem4AaSgabaGaeiikaGIaemOyaiMaeiykaKcaaaaa@3522@, we expect the mean score U¯(b)
 MathType@MTEF@5@5@+=feaafiart1ev1aaatCvAUfKttLearuWrP9MDH5MBPbIqV92AaeXatLxBI9gBaebbnrfifHhDYfgasaacH8akY=wiFfYdH8Gipec8Eeeu0xXdbba9frFj0=OqFfea0dXdd9vqai=hGuQ8kuc9pgc9s8qqaq=dirpe0xb9q8qiLsFr0=vr0=vr0dc8meaabaqaciaacaGaaeqabaqabeGadaaakeaacuWGvbqvgaqeamaaCaaaleqabaGaeiikaGIaemOyaiMaeiykaKcaaaaa@3123@ to be larger than U¯(a)
 MathType@MTEF@5@5@+=feaafiart1ev1aaatCvAUfKttLearuWrP9MDH5MBPbIqV92AaeXatLxBI9gBaebbnrfifHhDYfgasaacH8akY=wiFfYdH8Gipec8Eeeu0xXdbba9frFj0=OqFfea0dXdd9vqai=hGuQ8kuc9pgc9s8qqaq=dirpe0xb9q8qiLsFr0=vr0=vr0dc8meaabaqaciaacaGaaeqabaqabeGadaaakeaacuWGvbqvgaqeamaaCaaaleqabaGaeiikaGIaemyyaeMaeiykaKcaaaaa@3121@ when the associated marker is the QTL, or is linked in disequilibrium with it. To compare the two means, we may apply a one-sided paired t-test. Let U^ijk(a)=Uijk(a)−U¯(a),U^ijk(b)=Uijk(b)−U¯(b)
 MathType@MTEF@5@5@+=feaafiart1ev1aaatCvAUfKttLearuWrP9MDH5MBPbIqV92AaeXatLxBI9gBaebbnrfifHhDYfgasaacH8akY=wiFfYdH8Gipec8Eeeu0xXdbba9frFj0=OqFfea0dXdd9vqai=hGuQ8kuc9pgc9s8qqaq=dirpe0xb9q8qiLsFr0=vr0=vr0dc8meaabaqaciaacaGaaeqabaqabeGadaaakeaacuWGvbqvgaqcamaaDaaaleaacqWGPbqAcqWGQbGAcqWGRbWAaeaacqGGOaakcqWGHbqycqGGPaqkaaGccqGH9aqpcqWGvbqvdaqhaaWcbaGaemyAaKMaemOAaOMaem4AaSgabaGaeiikaGIaemyyaeMaeiykaKcaaOGaeyOeI0IafmyvauLbaebadaahaaWcbeqaaiabcIcaOiabdggaHjabcMcaPaaakiabcYcaSiqbdwfavzaajaWaa0baaSqaaiabdMgaPjabdQgaQjabdUgaRbqaaiabcIcaOiabdkgaIjabcMcaPaaakiabg2da9iabdwfavnaaDaaaleaacqWGPbqAcqWGQbGAcqWGRbWAaeaacqGGOaakcqWGIbGycqGGPaqkaaGccqGHsislcuWGvbqvgaqeamaaCaaaleqabaGaeiikaGIaemOyaiMaeiykaKcaaaaa@5C84@ and let *n *be the total number of sibpairs. The statistic is then defined by

T=(U¯(b)−U¯(a))n(n−1)/∑(U^ijk(b)−U^ijk(a))2     (5)
 MathType@MTEF@5@5@+=feaafiart1ev1aaatCvAUfKttLearuWrP9MDH5MBPbIqV92AaeXatLxBI9gBaebbnrfifHhDYfgasaacH8akY=wiFfYdH8Gipec8Eeeu0xXdbba9frFj0=OqFfea0dXdd9vqai=hGuQ8kuc9pgc9s8qqaq=dirpe0xb9q8qiLsFr0=vr0=vr0dc8meaabaqaciaacaGaaeqabaqabeGadaaakeaacqWGubavcqGH9aqpcqGGOaakcuWGvbqvgaqeamaaCaaaleqabaGaeiikaGIaemOyaiMaeiykaKcaaOGaeyOeI0IafmyvauLbaebadaahaaWcbeqaaiabcIcaOiabdggaHjabcMcaPaaakiabcMcaPmaakaaabaGaemOBa4MaeiikaGIaemOBa4MaeyOeI0IaeGymaeJaeiykaKIaei4la8YaaabqaeaacqGGOaakcuWGvbqvgaqcamaaDaaaleaacqWGPbqAcqWGQbGAcqWGRbWAaeaacqGGOaakcqWGIbGycqGGPaqkaaaabeqab0GaeyyeIuoakiabgkHiTiqbdwfavzaajaWaa0baaSqaaiabdMgaPjabdQgaQjabdUgaRbqaaiabcIcaOiabdggaHjabcMcaPaaakiabcMcaPmaaCaaaleqabaGaeGOmaidaaaqabaGccaWLjaGaaCzcamaabmaabaGaeGynaudacaGLOaGaayzkaaaaaa@5C85@

and under the null hypothesis follows a *t *distribution with degrees of freedom *n *- 1. The one sided p-value is given by *P*(*t*_*n *- 1 _> *T*).

It is useful to examine this statistic under various situations. When the marker modeled is not associated with the phenotype, the allelic effect *b *is expected to be small and therefore the statistic is likely to be close to zero. However, when there is an association between the marker and the quantitative trait in a statistical sense, but it is not related to the detected linkage (for example it is due to the well-known bias from population stratification), we may not expect the allelic effect *b *to be small. In this scenario, we may look upon the marker as a covariate representing to some extent population stratification, and therefore modeling this marker would reduce the residual variance of the trait similarity measure coming from population stratification, and hence strengthen the linkage signal. So we can expect the statistic *T *to be more likely to be negative, and our test statistic would maintain the type I error rate in a conservative fashion in the case of population stratification. Our simulation results agree with this line of reasoning (see results). In this sense, a small lower sided p-value, i.e. *P*(*t*_*n *- 1 _<*T*), indicates a spurious association, which is also seen in the simulations.

For simplicity, assume the allelic effect *b *and the sibship mean are *μ_k _*known and so can be specified correctly; it can then be easily shown that for sibpair (*i,j*) in family *k*, E(Uijk(b)−Uijk(a))=(xikxjkb2)IBDijk
 MathType@MTEF@5@5@+=feaafiart1ev1aaatCvAUfKttLearuWrP9MDH5MBPbIqV92AaeXatLxBI9gBaebbnrfifHhDYfgasaacH8akY=wiFfYdH8Gipec8Eeeu0xXdbba9frFj0=OqFfea0dXdd9vqai=hGuQ8kuc9pgc9s8qqaq=dirpe0xb9q8qiLsFr0=vr0=vr0dc8meaabaqaciaacaGaaeqabaqabeGadaaakeaacqWGfbqrcqGGOaakcqWGvbqvdaqhaaWcbaGaemyAaKMaemOAaOMaem4AaSgabaGaeiikaGIaemOyaiMaeiykaKcaaOGaeyOeI0Iaemyvau1aa0baaSqaaiabdMgaPjabdQgaQjabdUgaRbqaaiabcIcaOiabdggaHjabcMcaPaaakiabcMcaPiabg2da9iabcIcaOiabdIha4naaBaaaleaacqWGPbqAcqWGRbWAaeqaaOGaemiEaG3aaSbaaSqaaiabdQgaQjabdUgaRbqabaGccqWGIbGydaahaaWcbeqaaiabikdaYaaakiabcMcaPiabdMeajjabdkeacjabdseaenaaBaaaleaacqWGPbqAcqWGQbGAcqWGRbWAaeqaaaaa@56DA@ (see Appendix 2). This equation indicates that the proposed statistic essentially tests the correlation (or interaction) between the similarity of an associated marker effect, which is measured by a cross-product, and the IBD sharing between two sibs in a pair. Compared to a usual quantitative linkage analysis that detects linkage by testing the correlation between the IBD sharing and trait similarity, which may also be described as a cross-product (e.g. as in HE regressions and the variance component model), we can expect the proposed statistic to be much more powerful for detecting linkage because the noise (residual variances) from polygenic and common environmental effects is eliminated as well as the individual random effects. So, even if a usual linkage analysis fails to show signals in a region, the proposed statistic can still be useful to detect linkage when we have a candidate locus in a region.

## Results

### Sample selection

Because in practice the number of alleles shared IBD is generally not known with certainty, owing to partially informative markers and missing parental genotypes, we also performed computer simulations to examine the usefulness of the QLS in sample selection for an association study by comparing, in various situations, the statistics from random samples of unrelated individuals and from samples based on the rank order of the QLS. The statistic used to make the comparison is the score statistic proposed by Schaid et al. [19], which follows a *χ*^2 ^distribution with one degree of freedom for an additive model.

In our simulations, we generate 1000 sibships of size 2 from different subpopulations. A total of 6 markers, evenly space at a 2 cM density in a 10 cM range and each with 4 equally frequent alleles, are used for the linkage analysis. A QTL with 2 equally frequent alleles is located midway between marker 3 and marker 4. We assume Hardy-Weinberg equilibrium at each marker, linkage equilibrium among the markers and a Haldane no-interference map function. Trait values are constructed as the sum of a major-gene effect generated by the QTL, normal random individual effects, polygenic effects and common environmental effects. We calculate the probabilities of the number of alleles shared IBD using the program GENIBD in the S.A.G.E. package [20], removing the QTL genotype for this calculation.

**Figure 2:**
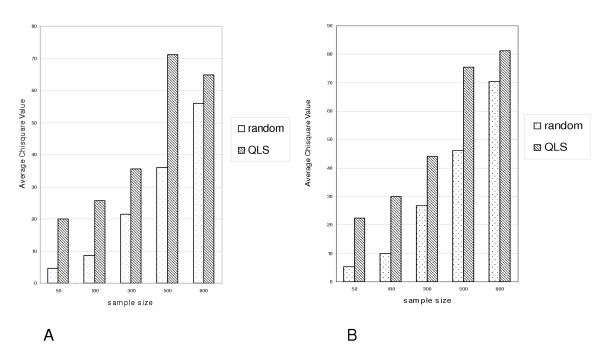
**Comparison of average *χ*^2 ^values between random sampling and QLS sampling for various sample sizes**. A. Two subpopulations: 20% of total variance is from an additive QTL in subpopulation 1, and no QTL effect exists in subpopulation 2. B. Four subpopulations with the QTL effect explaining 0%, 5%, 10%, and 20% of the total variance.

We first compare random selection and the QLS selection with different sample sizes for the association study. We assume the population consists of two subpopulations, in equal proportions, from which 1,000 sibpairs have been used for the linkage analysis. In subpopulation 1, 20% of the total variance is explained by the QTL, 30% by the polygenic and common environmental effects and the rest by a random individual effect. In subpopulation 2, there is no QTL effect but the same other effects are simulated. We separately sample 50, 100, 300, 500 and 800 unrelated individuals from the 1000 sibpairs by the two selection approaches and compare their score statistics. In QLS selection, we first select sibships with largest QLS and then randomly select one sib from each of these pairs, while in random selection the sibpair is selected randomly. The average *χ*^2 ^is shown in Figure 2(A). In real data, the situation may be more complex in that a population may consist of more than two subpopulations and the QTL effect could vary among subpopulations. We therefore also simulated four subpopulations with equal proportions having different QTL effects (0%, 5%, 10%, 20%) and compared the association statistics for different sample sizes. The results are shown in Figure 2(B). In both Scenarios (A and B), QLS selection can greatly increase the average value of the statistic to detect association, and this increase is larger when fewer unrelated individuals are selected.

To examine different ways of summarizing the several QLSs for a sibship, we also simulated sibships of different sizes, ranging from 2 to 4. The traits for the population with two subpopulations were simulated as before. We sampled 100 unrelated individuals from the 1000 sibships at random, or according to the rank order of the mean QLS, the minimum QLS and the maximum QLS of each sibship, respectively. Our results showed that the average *χ*^2 ^values obtained based on any of the QLSs are greater than those from random selection and that they have small differences between them (χmean2>χmax2>χmin2
 MathType@MTEF@5@5@+=feaafiart1ev1aaatCvAUfKttLearuWrP9MDH5MBPbIqV92AaeXatLxBI9gBaebbnrfifHhDYfgasaacH8akY=wiFfYdH8Gipec8Eeeu0xXdbba9frFj0=OqFfea0dXdd9vqai=hGuQ8kuc9pgc9s8qqaq=dirpe0xb9q8qiLsFr0=vr0=vr0dc8meaabaqaciaacaGaaeqabaqabeGadaaakeaaiiGacqWFhpWydaqhaaWcbaGaemyBa0MaemyzauMaemyyaeMaemOBa4gabaGaeGOmaidaaOGaeyOpa4Jae83Xdm2aa0baaSqaamXvP5wqSXMqHnxAJn0BKvguHDwzZbqegyvzYrwyUfgaiqGacaGFTbGaa4xyaiaa+HhaaeaacqaIYaGmaaGccqGH+aGpcqWFhpWydaqhaaWcbaGaa4xBaiaa+LgacaGFUbaabaGaeGOmaidaaaaa@4CCC@) (data not shown).

**Figure 3:**
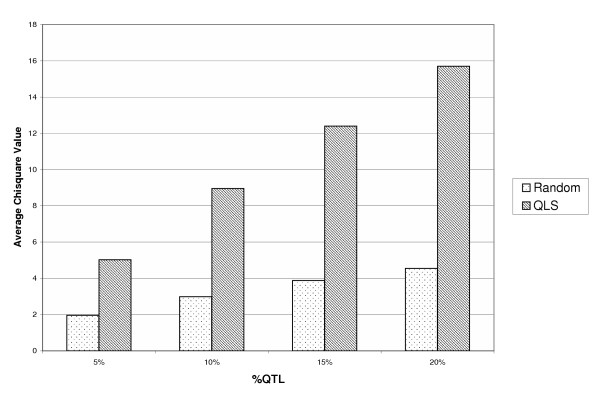
Comparison of average *χ*^2 ^values between random sampling and QLS sampling when the power to detect linkage is small.

Although in this paper we focus on the usefulness of the QLS in the situation where a significant linkage region has already been identified, we are also interested in the situation where the linkage signal is not so clear, because in the case of a complex quantitative trait we expect only weak linkage signals when using customary sample sizes. To show the usefulness of QLS selection in this scenario, we also simulated 500 sibpairs from two subpopulations in which different proportions of the variance (5%, 10%, 15%, and 20%) are explained by the QTL in just one subpopulation. In this simulation, linkage signals are quite small and even cannot be detected. We sampled 100 unrelated individuals for an association study. The results show that the sampling based on the QLS still improves the power of an association study, even in the case that the power to detect linkage is negligible (see Figure 3).

**Figure 4:**
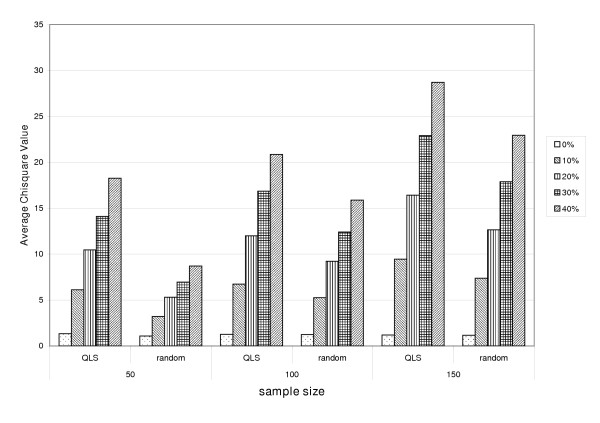
**Comparison of average *χ*^2 ^values between random sampling and QLS sampling for a joint linkage/association method**. Two subpopulations: 0%, 10%, 20%, 30% and 40% of total variance is from an additive QTL in subpopulation 1, and no QTL effect exists in subpopulation 2.

At the stage of the association study, a family sample is also often used and then a joint linkage/association analysis can be applied in this case. One advantage of the joint linkage/assocation model is that, when it detects association, this method can simultaneously take account of the linkage information. We also performed a simulation study to examine the usefulness of QLS based sample selection in this case. A total of 500 nuclear families of size 4 from two subpopulations, in equal proportion, were generated for the previous linkage study. We further sampled 50, 100 and 150 families for fining mapping. Different QTL effects were simulated in subpopulation 1 (0%, 10%, 20%, 30% and 40%) and subpopulation 2 (0%). We compared the statistics of a commonly used joint linkage/association method (awbw) for a random sample and QLS based sample of families [21]. The results show the power of this joint analysis can also be greatly improved by the QLS selection approach (see Figure 4).

### Testing the correlation between association and a previous linkage

To assess the properties of our tests to determine whether an association is responsible in part for the linkage of a complex quantitative trait, we carried out a limited simulation study. We examined the type I error rate of the proposed test under two scenarios: (1) no trait-marker association and (2) trait-marker association due to population stratification. Under no trait-marker association, we simulated 10,000 replicate data sets of 500 sibpairs or 500 sibships (200, 200 and 100 sibships of sizes 2, 3 and 4, respectively). Trait values were constructed as the sum of a major-gene effect generated by the QTL that explains 10% of the variance, and various proportions of random individual, polygenic and common environmental effects. An association marker with two equally frequent alleles was simulated to be in complete linkage equilibrium with the QTL and a fully informative linkage marker (with 100 equal frequent alleles) was also simulated at the same location. For the case of linkage but no trait-marker association, the results show that the type I error rate of the proposed statistic is generally good for a complex quantitative trait for both sibpair data and sibship data (see Table 1). Under a spurious association, we generated 10,000 replicate datasets of 500 sibpairs from two subpopulations. The trait mean and the frequencies of the marker alleles were different in the two subpopulations. The results (see Table 2) show that the power to detect linkage is consistent in various situations suggesting that the linkage test is quite robust to population stratification (the"linkage" column). For the proposed test examining the linkage-association correlation, the type I error rate is controlled conservatively (the "association-linakge" colmun). When the effect of population stratification is small, the empirical type I error rate is close to the correct level (0.05). We also examined the usefulness of the proposed statistic for detecting spurious association due to population stratification by using the lower sided t-test (shown in the "population stratification" column of Table 2). The results suggest that in practice when the association cannot explain any of the linkage, this statistic may nevertheless be useful to determine whether the association is "false".

**Table 1:** Empirical type I error rate of the proposed test at the nominal 5% level. A diallelic marker is completely linked to the QTL under HW equilibrium.

Effects ^1^	500 sibpairs		500 sibships ^2^	
		
	Linkage	Association-Linkage	Linkage	Association-Linkage
10%/80%	0.195	0.056	0.391	0.059
20%/70%	0.211	0.059	0.310	0.057
30%/60%	0.236	0.055	0.328	0.055
40%/50%	0.248	0.054	0.346	0.058
50%/40%	0.270	0.056	0.368	0.056

^1 ^Common environmental & polygenic effects/individual random effects

^2^There are 200 sibships of size 2, 200 sibships of size 3 and 100 sibships of size 4.

**Table 2:** Empirical type I error rate of the proposed test at the nominal 5% level when population stratification exists. A diallelic marker is completely linked to the QTL with HW equilibrium in an admixed population. Total 500 sibpairs (250/250) are selected from two subpopulations. *p*_1 _and *p*_1 _are frequencies of the rarer marker allele in the two subpopulations. *d *is the difference in trait means between two subpopulations.

*p*_1 _- *p*_2_	*d *= 10			*d *= 20		
		
	Linkage	Association-Linkage	Population stratification	Linkage	Association-Linkage	Population stratification
0.4	0.200	0.00055	0.403	0.165	0	0.758
0.3	0.191	0.0016	0.311	0.166	0	0.536
0.2	0.200	0.0054	0.177	0.159	0.001	0.283
0.1	0.192	0.025	0.084	0.154	0.012	0.106
0	0.194	0.050	0.019	0.150	0.047	0.013

We also performed simulations to assess the power of the proposed statistic to detect the correlation between the gene effect and IBD sharing. We compared this statistic with a revised HE regression that we have shown is one of the most powerful versions of HE [11]. An associated marker with two equal allele frequencies was simulated as the QTL itself. We generated trait values with various different QTL effects, keeping fixed polygenic, common environmental effects and individual random effects. We considered two sets of linkage markers: fully informative and partially informative (six markers were used for the linkage analysis, evenly spaced at a 2 cM density in a 10 cM range around the QTL). For each situation, we generated 1,000 replicate samples of data on 500 sibpairs. Table 3 shows that by incorporating information on the candidate marker the proposed test is much more powerful than quantitative linkage analysis. In general, for a complex quantitative trait, usual linkage analysis may lack power and therefore miss an important region, because the noise from other genetic and environmental effects masks the linked gene effect. When no linkage is detected in a region where an important candidate gene is located, it is not wise to discard this region from further study. We may use the proposed statistic to assess whether the "negative" linkage result is true.

**Table 3:** The power of the proposed test for 500 sibpairs when linkage signal is weak. A diallelic marker is completely linked to the QTL in perfect disequilibrium. The trait value is generated by a QTL with variance of varying size, together with polygenic and common environmental effects (with variance 0.3) and a random individual effect (with variance 0.5).

QTL variance	Fully informative marker		Partially informative marker	
		
	Linkage	Association-Linkage	Linkage	Association-Linkage
0.03	0.102	0.307	0.083	0.285
0.05	0.148	0.402	0.145	0.475
0.10	0.284	0.666	0.260	0.575
0.20	0.563	0.874	0.520	0.838

## Discussion

There is great interest in QTL mapping because many important diseases themselves, or intermediate phenotypes, are measured on a continuous scale. Although trait-marker association studies are expected to be soon conducted genome-wide, because of cost considerations currently an association study often focuses on candidate regions determined by a previous linkage study. For such an association study, we should utilize the information available in the previous linkage study to optimize its design and to facilitate its interpretation. We have proposed a quantitative linkage score, based on the widely used HE regression, to provide quantitative linkage information useful for a follow-up association study. This score is not limited to continuous traits, but can also be used for binary (affected/unaffected) traits. We illustrated the usefulness of this score to answer two different questions posed by an association study: (1) how to select samples at the design stage when heterogeneity exists; and (2) how to test at the inference stage whether an observed association can explain in part a previous linkage signal. In this paper, we are not necessarily advocating a two-stage approach to analyze family data on which we have information on both linkage markers and association markers. For such data a joint linkage and association framework could be of more interest than a two-stage analysis approach. Recent work on this kind of joint analysis has included work on both regression-based methods [22] and variance-component methods [23,24]. However, in the presence of heterogeneity any advantage such a joint analysis may have when performed using all the data available may be lost, because those families that are not affected because of segregation at a linked locus will "dilute" the effect and result in loss of power. Therefore, even for analyzing data with information from both linkage markers and association markers, we may consider first selecting families based on the QLS to exclude such "dilution" as much as possible.

The idea of selecting families with linkage evidence for further genotyping in a follow-up association study is not new and has been successfully implemented in practice. In the context of quantitative traits, the proposed score can conveniently be used to summarize quantitative linkage information from a sibpair (or sibship). We have shown that in a heterogeneous population, which is expected to commonly occur for a complex trait, selecting a sample of unrelated persons based on the order of the QLS magnitude results in a more homogeneous sample for an association study than does a random sample, and therefore can improve power for a given sample size. Other approaches to identifying sibpairs with linkage are available, for example using a regression diagnostic [25]. Careful comparison of these methods would merit further study.

Another use of the QLS investigated in this paper is to test whether association can account in part for a detected linkage. To address this question, we simply compare two sets of QLSs, before and after incorporating an association marker into the individual level regression model. Essentially, the proposed test evaluates the interaction of the allele effect of an associated marker and IBD sharing. In this sense it may be likened to other methods, for example the regression model proposed by Cardon [26], though our statistic emphasizes more whether an association is correlated with a previous linkage finding. This test may also be used as a substitute for the usual quantitative trait linkage analysis test when the latter fails to detect linkage. The gain in power to detect linkage by using the proposed test arises from eliminating possible environmental or other genetic noise. However, this gain is not automatic, but depends on the relationship of the associated marker to the true variant. If there is only weak linkage disequilibrium between an associated marker and the true variant, the test will be less powerful. We also showed that this statistic may be applied to detect spurious association, although that was not our primary aim. The ways commonly used in practice to detect population stratification are to use genomic control [27] or test for Hardy-Weinberg equilibrium [28]. Using IBD sharing information to test and control for population stratification provides a new approach and further study of this approach will be conducted in our future work.

## Conclusion

In conclusion, as proved by our simulations, the QLS is useful for the design of, and resulting inference from, an association study following a linkage study. We suggest that careful examination of the QLS should be helpful for understanding the results of both association and linkage studies.

## Authors' contributions

TW contributed to the conception of the study, performed the simulation analysis, and wrote the manuscript. RCE contributed to the conception of the study and the writing of the manuscript.

## Appendix

**Appendix 1 the derivation of ***q_qls_*

For sibpair *k *comprising sib 1 and sib 2, *Z*_*k*_(*z*_1*k*_, *z*_2*k*_) follows the distribution *f *(*z*_1*k*_, *z*_2*k*_), which we assume to be a bivariate normal distribution. With the assumption that a random sample of full sibpairs is used for the linkage analysis, the proportions of pairs for which the number of alleles shared IBD is 0, 1 and 2 are *π*_0 _= 1/4, *π*_1 _= 1/2 and *π*_2 _= 1/4, respectively. Let P1 and P2 refer to subpopulation 1 and 2 and let their proportions be denoted *q*_1 _and *q*_2_. We then have

Pr(QLS>0|P1)=2Pr(z1k>0,z2k>0,IBD=1|P1)+2Pr(z1k>0,z2k<0,IBD=0|P1)=2π2∫0∞∫0∞f(z1k,z2k|IBD=1,P1)dz1kdz2k+2π0∫0∞∫−∞0f(z1k,z2k|IBD=0,P1)dz1kdz2k=14[1π arctan (ρIBD=121−ρIBD=12)+1].
 MathType@MTEF@5@5@+=feaafiart1ev1aaatCvAUfKttLearuWrP9MDH5MBPbIqV92AaeXatLxBI9gBaebbnrfifHhDYfgasaacH8akY=wiFfYdH8Gipec8Eeeu0xXdbba9frFj0=OqFfea0dXdd9vqai=hGuQ8kuc9pgc9s8qqaq=dirpe0xb9q8qiLsFr0=vr0=vr0dc8meaabaqaciaacaGaaeqabaqabeGadaaakeaafaqaaeWadaaabaWexLMBbXgBcf2CPn2qVrwzqf2zLnharyGvLjhzH5wyaGabciaa=bfacaWFYbGaeiikaGIaemyuaeLaemitaWKaem4uamLaeyOpa4JaeGimaaJaeiiFaWNaemiuaaLaeGymaeJaeiykaKcabaGaeyypa0dabaGaeGOmaiJaa8huaiaa=jhacqGGOaakcqWG6bGEdaWgaaWcbaGaeGymaeJaem4AaSgabeaakiabg6da+iabicdaWiabcYcaSiabdQha6naaBaaaleaacqaIYaGmcqWGRbWAaeqaaOGaeyOpa4JaeGimaaJaeiilaWIaemysaKKaemOqaiKaemiraqKaeyypa0JaeGymaeJaeiiFaWNaemiuaaLaeGymaeJaeiykaKIaey4kaSIaeGOmaiJaa8huaiaa=jhacqGGOaakcqWG6bGEdaWgaaWcbaGaeGymaeJaem4AaSgabeaakiabg6da+iabicdaWiabcYcaSiabdQha6naaBaaaleaacqaIYaGmcqWGRbWAaeqaaOGaeyipaWJaeGimaaJaeiilaWIaemysaKKaemOqaiKaemiraqKaeyypa0JaeGimaaJaeiiFaWNaemiuaaLaeGymaeJaeiykaKcabaaabaGaeyypa0dabaGaeGOmaidcciGae4hWda3aaSbaaSqaaiabikdaYaqabaGcdaWdXaqaamaapedabaGaemOzayMaeiikaGYaaqGaaeaacqWG6bGEdaWgaaWcbaGaeGymaeJaem4AaSgabeaakiabcYcaSiabdQha6naaBaaaleaacqaIYaGmcqWGRbWAaeqaaaGccaGLiWoacqWGjbqscqWGcbGqcqWGebarcqGH9aqpcqaIXaqmcqGGSaalcqWGqbaucqaIXaqmcqGGPaqkcqWGKbazdaWgaaWcbaGaemOEaO3aaSbaaWqaaiabigdaXiabdUgaRbqabaaaleqaaOGaemizaq2aaSbaaSqaaiabdQha6naaBaaameaacqaIYaGmcqWGRbWAaeqaaaWcbeaakiabgUcaRiabikdaYiab+b8aWnaaBaaaleaacqaIWaamaeqaaOWaa8qmaeaadaWdXaqaaiabdAgaMjabcIcaOmaaeiaabaGaemOEaO3aaSbaaSqaaiabigdaXiabdUgaRbqabaGccqGGSaalcqWG6bGEdaWgaaWcbaGaeGOmaiJaem4AaSgabeaaaOGaayjcSdGaemysaKKaemOqaiKaemiraqKaeyypa0JaeGimaaJaeiilaWIaemiuaaLaeGymaeJaeiykaKIaemizaq2aaSbaaSqaaiabdQha6naaBaaameaacqaIXaqmcqWGRbWAaeqaaaWcbeaakiabdsgaKnaaBaaaleaacqWG6bGEdaWgaaadbaGaeGOmaiJaem4AaSgabeaaaSqabaaabaGaeyOeI0IaeyOhIukabaGaeGimaadaniabgUIiYdaaleaacqaIWaamaeaacqGHEisPa0Gaey4kIipaaSqaaiabicdaWaqaaiabg6HiLcqdcqGHRiI8aaWcbaGaeGimaadabaGaeyOhIukaniabgUIiYdaakeaaaeaacqGH9aqpaeaadaWcaaqaaiabigdaXaqaaiabisda0aaadaWadaqaamaalaaabaGaeGymaedabaGae4hWdahaaiabbccaGiabbggaHjabbkhaYjabbogaJjabbsha0jabbggaHjabb6gaUjabbccaGmaabmaabaWaaSaaaeaacqGFbpGCdaqhaaWcbaGaemysaKKaemOqaiKaemiraqKaeyypa0JaeGymaedabaGaeGOmaidaaaGcbaGaeGymaeJaeyOeI0Iae4xWdi3aa0baaSqaaiabdMeajjabdkeacjabdseaejabg2da9iabigdaXaqaaiabikdaYaaaaaaakiaawIcacaGLPaaacqGHRaWkcqaIXaqmaiaawUfacaGLDbaacqGGUaGlaaaaaa@012C@

Thus,

Pr(QLS>0,P1)=Pr(QlS>0|P1)Pr(P1)=14q1[1π arctan (ρIBD=121−ρIBD=12)+1],
 MathType@MTEF@5@5@+=feaafiart1ev1aaatCvAUfKttLearuWrP9MDH5MBPbIqV92AaeXatLxBI9gBaebbnrfifHhDYfgasaacH8akY=wiFfYdH8Gipec8Eeeu0xXdbba9frFj0=OqFfea0dXdd9vqai=hGuQ8kuc9pgc9s8qqaq=dirpe0xb9q8qiLsFr0=vr0=vr0dc8meaabaqaciaacaGaaeqabaqabeGadaaakeaafaqaaeGadaaabaWexLMBbXgBcf2CPn2qVrwzqf2zLnharyGvLjhzH5wyaGabciaa=bfacaWFYbGaeiikaGIaemyuaeLaemitaWKaem4uamLaeyOpa4JaeGimaaJaeiilaWIaemiuaaLaeGymaeJaeiykaKcabaGaeyypa0dabaGaa8huaiaa=jhacqGGOaakcqWGrbqucqWGSbaBcqWGtbWucqGH+aGpcqaIWaamcqGG8baFcqWGqbaucqaIXaqmcqGGPaqkcaWFqbGaa8NCaiabcIcaOiabdcfaqjabigdaXiabcMcaPaqaaaqaaiabg2da9aqaamaalaaabaGaeGymaedabaGaeGinaqdaaiabdghaXnaaBaaaleaacqaIXaqmaeqaaOWaamWaaeaadaWcaaqaaiabigdaXaqaaGGaciab+b8aWbaacqqGGaaicqqGHbqycqqGYbGCcqqGJbWycqqG0baDcqqGHbqycqqGUbGBcqqGGaaidaqadaqaamaalaaabaGae4xWdi3aa0baaSqaaiabdMeajjabdkeacjabdseaejabg2da9iabigdaXaqaaiabikdaYaaaaOqaaiabigdaXiabgkHiTiab+f8aYnaaDaaaleaacqWGjbqscqWGcbGqcqWGebarcqGH9aqpcqaIXaqmaeaacqaIYaGmaaaaaaGccaGLOaGaayzkaaGaey4kaSIaeGymaedacaGLBbGaayzxaaGaeiilaWcaaaaa@80B8@

which is an increasing function of *q*_1 _and *ρ*. We note that *ρ *depends on the size of the effect and allelic frequencies of the QTL. On the other hand,

Pr(QLS>0|P2)=2Pr(z1k>0,z2k>0,IBD=1|P2)+2Pr(z1k>0,z2k<0,IBD=0|P2)=14,
 MathType@MTEF@5@5@+=feaafiart1ev1aaatCvAUfKttLearuWrP9MDH5MBPbIqV92AaeXatLxBI9gBaebbnrfifHhDYfgasaacH8akY=wiFfYdH8Gipec8Eeeu0xXdbba9frFj0=OqFfea0dXdd9vqai=hGuQ8kuc9pgc9s8qqaq=dirpe0xb9q8qiLsFr0=vr0=vr0dc8meaabaqaciaacaGaaeqabaqabeGadaaakeaafaqaaeGadaaabaWexLMBbXgBcf2CPn2qVrwzqf2zLnharyGvLjhzH5wyaGabciaa=bfacaWFYbGaeiikaGIaemyuaeLaemitaWKaem4uamLaeyOpa4JaeGimaaJaeiiFaWNaemiuaaLaeGOmaiJaeiykaKcabaGaeyypa0dabaGaeGOmaiJaa8huaiaa=jhacqGGOaakcqWG6bGEdaWgaaWcbaGaeGymaeJaem4AaSgabeaakiabg6da+iabicdaWiabcYcaSiabdQha6naaBaaaleaacqaIYaGmcqWGRbWAaeqaaOGaeyOpa4JaeGimaaJaeiilaWIaemysaKKaemOqaiKaemiraqKaeyypa0JaeGymaeJaeiiFaWNaemiuaaLaeGOmaiJaeiykaKIaey4kaSIaeGOmaiJaa8huaiaa=jhacqGGOaakcqWG6bGEdaWgaaWcbaGaeGymaeJaem4AaSgabeaakiabg6da+iabicdaWiabcYcaSiabdQha6naaBaaaleaacqaIYaGmcqWGRbWAaeqaaOGaeyipaWJaeGimaaJaeiilaWIaemysaKKaemOqaiKaemiraqKaeyypa0JaeGimaaJaeiiFaWNaemiuaaLaeGOmaiJaeiykaKcabaaabaGaeyypa0dabaWaaSaaaeaacqaIXaqmaeaacqaI0aanaaGaeiilaWcaaaaa@7F53@

so that *Pr*(*QLS *> 0, *P*2) = *Pr*(*QlS *> 0|*P*2)*Pr*(*P*2) = 14q2
 MathType@MTEF@5@5@+=feaafiart1ev1aaatCvAUfKttLearuWrP9MDH5MBPbIqV92AaeXatLxBI9gBaebbnrfifHhDYfgasaacH8akY=wiFfYdH8Gipec8Eeeu0xXdbba9frFj0=OqFfea0dXdd9vqai=hGuQ8kuc9pgc9s8qqaq=dirpe0xb9q8qiLsFr0=vr0=vr0dc8meaabaqaciaacaGaaeqabaqabeGadaaakeaadaWcaaqaaiabigdaXaqaaiabisda0aaacqWGXbqCdaWgaaWcbaGaeGOmaidabeaaaaa@312B@.

Thus

qqls=Pr(QLS>0,P1)Pr(QLS>0,P1)+Pr(QLS>0,P2)
 MathType@MTEF@5@5@+=feaafiart1ev1aaatCvAUfKttLearuWrP9MDH5MBPbIqV92AaeXatLxBI9gBaebbnrfifHhDYfgasaacH8akY=wiFfYdH8Gipec8Eeeu0xXdbba9frFj0=OqFfea0dXdd9vqai=hGuQ8kuc9pgc9s8qqaq=dirpe0xb9q8qiLsFr0=vr0=vr0dc8meaabaqaciaacaGaaeqabaqabeGadaaakeaacqWGXbqCdaWgaaWcbaGaemyCaeNaemiBaWMaem4Camhabeaakiabg2da9maalaaabaWexLMBbXgBcf2CPn2qVrwzqf2zLnharyGvLjhzH5wyaGabciaa=bfacaWFYbGaeiikaGIaemyuaeLaemitaWKaem4uamLaeyOpa4JaeGimaaJaeiilaWIaemiuaaLaeGymaeJaeiykaKcabaGaa8huaiaa=jhacqGGOaakcqWGrbqucqWGmbatcqWGtbWucqGH+aGpcqaIWaamcqGGSaalcqWGqbaucqaIXaqmcqGGPaqkcqGHRaWkcaWFqbGaa8NCaiabcIcaOiabdgfarjabdYeamjabdofatjabg6da+iabicdaWiabcYcaSiabdcfaqjabikdaYiabcMcaPaaaaaa@62AF@

=11+q21π arctan (ρIBD=121−ρIBD=12+1).
 MathType@MTEF@5@5@+=feaafiart1ev1aaatCvAUfKttLearuWrP9MDH5MBPbIqV92AaeXatLxBI9gBaebbnrfifHhDYfgasaacH8akY=wiFfYdH8Gipec8Eeeu0xXdbba9frFj0=OqFfea0dXdd9vqai=hGuQ8kuc9pgc9s8qqaq=dirpe0xb9q8qiLsFr0=vr0=vr0dc8meaabaqaciaacaGaaeqabaqabeGadaaakeaacqGH9aqpdaWcaaqaaiabigdaXaqaaiabigdaXiabgUcaRmaalaaabaGaemyCae3aaSbaaSqaaiabikdaYaqabaaakeaadaWcbaWcbaGaeGymaedabaacciGae8hWdahaaOGaeeiiaaIaeeyyaeMaeeOCaiNaee4yamMaeeiDaqNaeeyyaeMaeeOBa4MaeeiiaaYaaeWaaeaadaWcbaWcbaGae8xWdi3aa0baaWqaaiabdMeajjabdkeacjabdseaejabg2da9iabigdaXaqaaiabikdaYaaaaSqaaiabigdaXiabgkHiTiab=f8aYnaaDaaameaacqWGjbqscqWGcbGqcqWGebarcqGH9aqpcqaIXaqmaeaacqaIYaGmaaaaaOGaey4kaSIaeGymaedacaGLOaGaayzkaaaaaaaacqGGUaGlaaa@560C@

**Appendix 2 **- E(*U*^(*b*) ^- *U*^(*a*)^)

Under the trait model *y*_*ik *_= *μ_k _*+ *x*_*ik*_*b *+ *e*_*ik*_, we assume the *e*_*ik *_are identically and independently distributed with mean 0. Suppose *μ_k _*and *b *are known. Let the subscripts 1 and 2 indicate the two sibs of a sibpair in family *k*. Then

E(Uk(b)−Uk(a)=E[(y1k−μk)(y2k−μk)−(y1k−μk−x1kb)(y2k−μk−x2kb)]IBD12k=E[(y1k−μk)x2kb+(y2k−μk)x1kb−x1kx2kb2]IBD12k=E[x1kx2kb2+e1kx2kb+e2kx1kb]IBD12k=(x1kx2kb2)IBD12k
 MathType@MTEF@5@5@+=feaafiart1ev1aaatCvAUfKttLearuWrP9MDH5MBPbIqV92AaeXatLxBI9gBaebbnrfifHhDYfgasaacH8akY=wiFfYdH8Gipec8Eeeu0xXdbba9frFj0=OqFfea0dXdd9vqai=hGuQ8kuc9pgc9s8qqaq=dirpe0xb9q8qiLsFr0=vr0=vr0dc8meaabaqaciaacaGaaeqabaqabeGadaaakeaafaqaaeabdaaaaeaacqWGfbqrcqGGOaakcqWGvbqvdaqhaaWcbaGaem4AaSgabaGaeiikaGIaemOyaiMaeiykaKcaaOGaeyOeI0Iaemyvau1aa0baaSqaaiabdUgaRbqaaiabcIcaOiabdggaHjabcMcaPaaaaOqaaiabg2da9aqaaiabdweafjabcUfaBjabcIcaOiabdMha5naaBaaaleaacqaIXaqmcqWGRbWAaeqaaOGaeyOeI0ccciGae8hVd02aaSbaaSqaaiabdUgaRbqabaGccqGGPaqkcqGGOaakcqWG5bqEdaWgaaWcbaGaeGOmaiJaem4AaSgabeaakiabgkHiTiab=X7aTnaaBaaaleaacqWGRbWAaeqaaOGaeiykaKIaeyOeI0IaeiikaGIaemyEaK3aaSbaaSqaaiabigdaXiabdUgaRbqabaGccqGHsislcqWF8oqBdaWgaaWcbaGaem4AaSgabeaakiabgkHiTiabdIha4naaBaaaleaacqaIXaqmcqWGRbWAaeqaaOGaemOyaiMaeiykaKIaeiikaGIaemyEaK3aaSbaaSqaaiabikdaYiabdUgaRbqabaGccqGHsislcqWF8oqBdaWgaaWcbaGaem4AaSgabeaakiabgkHiTiabdIha4naaBaaaleaacqaIYaGmcqWGRbWAaeqaaOGaemOyaiMaeiykaKIaeiyxa0LaemysaKKaemOqaiKaemiraq0aaSbaaSqaaiabigdaXiabikdaYiabdUgaRbqabaaakeaaaeaacqGH9aqpaeaacqWGfbqrcqGGBbWwcqGGOaakcqWG5bqEdaWgaaWcbaGaeGymaeJaem4AaSgabeaakiabgkHiTiab=X7aTnaaBaaaleaacqWGRbWAaeqaaOGaeiykaKIaemiEaG3aaSbaaSqaaiabikdaYiabdUgaRbqabaGccqWGIbGycqGHRaWkcqGGOaakcqWG5bqEdaWgaaWcbaGaeGOmaiJaem4AaSgabeaakiabgkHiTiab=X7aTnaaBaaaleaacqWGRbWAaeqaaOGaeiykaKIaemiEaG3aaSbaaSqaaiabigdaXiabdUgaRbqabaGccqWGIbGycqGHsislcqWG4baEdaWgaaWcbaGaeGymaeJaem4AaSgabeaakiabdIha4naaBaaaleaacqaIYaGmcqWGRbWAaeqaaOGaemOyai2aaWbaaSqabeaacqaIYaGmaaGccqGGDbqxcqWGjbqscqWGcbGqcqWGebardaWgaaWcbaGaeGymaeJaeGOmaiJaem4AaSgabeaaaOqaaaqaaiabg2da9aqaaiabdweafjabcUfaBjabdIha4naaBaaaleaacqaIXaqmcqWGRbWAaeqaaOGaemiEaG3aaSbaaSqaaiabikdaYiabdUgaRbqabaGccqWGIbGydaahaaWcbeqaaiabikdaYaaakiabgUcaRiabdwgaLnaaBaaaleaacqaIXaqmcqWGRbWAaeqaaOGaemiEaG3aaSbaaSqaaiabikdaYiabdUgaRbqabaGccqWGIbGycqGHRaWkcqWGLbqzdaWgaaWcbaGaeGOmaiJaem4AaSgabeaakiabdIha4naaBaaaleaacqaIXaqmcqWGRbWAaeqaaOGaemOyaiMaeiyxa0LaemysaKKaemOqaiKaemiraq0aaSbaaSqaaiabigdaXiabikdaYiabdUgaRbqabaaakeaaaeaacqGH9aqpaeaacqGGOaakcqWG4baEdaWgaaWcbaGaeGymaeJaem4AaSgabeaakiabdIha4naaBaaaleaacqaIYaGmcqWGRbWAaeqaaOGaemOyai2aaWbaaSqabeaacqaIYaGmaaGccqGGPaqkcqWGjbqscqWGcbGqcqWGebardaWgaaWcbaGaeGymaeJaeGOmaiJaem4AaSgabeaaaaaaaa@EF05@
